# Insights into the mechanism of C5aR inhibition by PMX53 via implicit solvent molecular dynamics simulations and docking

**DOI:** 10.1186/2046-1682-7-5

**Published:** 2014-08-12

**Authors:** Phanourios Tamamis, Chris A Kieslich, Gregory V Nikiforovich, Trent M Woodruff, Dimitrios Morikis, Georgios Archontis

**Affiliations:** 1 Department of Physics, University of Cyprus, PO 20537, CY1678 Nicosia, Cyprus; 2 Department of Bioengineering, University of California, Riverside, CA 92521, USA; 3 MolLife Design LLC, St. Louis 63141, USA; 4 School of Biomedical Sciences, the University of Queensland, St Lucia 4072, Australia

**Keywords:** Class A GPCR, C5aR, C5a, Complement system, Molecular dynamics, Docking, Implicit solvent, Membrane protein

## Abstract

**Background:**

The complement protein C5a acts by primarily binding and activating the G-protein coupled C5a receptor C5aR (CD88), and is implicated in many inflammatory diseases. The cyclic hexapeptide PMX53 (sequence Ace-Phe-[Orn-Pro-dCha-Trp-Arg]) is a full C5aR antagonist of nanomolar potency, and is widely used to study C5aR function in disease.

**Results:**

We construct for the first time molecular models for the C5aR:PMX53 complex without the a priori use of experimental constraints, via a computational framework of molecular dynamics (MD) simulations, docking, conformational clustering and free energy filtering. The models agree with experimental data, and are used to propose important intermolecular interactions contributing to binding, and to develop a hypothesis for the mechanism of PMX53 antagonism.

**Conclusion:**

This work forms the basis for the design of improved C5aR antagonists, as well as for atomic-detail mechanistic studies of complement activation and function. Our computational framework can be widely used to develop GPCR-ligand structural models in membrane environments, peptidomimetics and other chemical compounds with potential clinical use.

## Background

C5aR is the membrane-bound receptor for the complement system protein C5a and the target of inhibition against inflammatory diseases. Here, we present the development of a structural model for the complex of membrane-embedded C5aR and its antagonist peptide PMX53. We accomplish this by an innovative and comprehensive computational framework that combines conformational sampling for both receptor and ligand with docking, and evaluates a large number (~300,000) of docked conformations by structural and free energy criteria. Even though we do not impose any experimental restraints, the resulting structural models are consistent with available experimental data.

The complement system is a major and essential component of the innate immune response. It can be activated following infection or injury through four distinct pathways, which lead to opsonisation of pathogens, cell lysis, and the production of potent pro-inflammatory peptides. The complement protein C5a is generated following cleavage of the 5^th^ component of complement (C5), and is one of the most potent inflammatory mediators in humans [1]. Given its potent inflammatory activity, prolonged or inappropriate activation of complement can generate unwanted C5a, which is implicated in many inflammatory diseases [2,3].

C5a induces the majority of its known effects primarily through a G-protein coupled receptor, termed C5aR. It can also bind to a second receptor called C5L2, however this receptor does not couple G-proteins, and thus has unclear and controversial functions [4]. The C-terminal ten residues of C5a have been shown to be critical for C5aR activation, and can induce full activation (efficacy) of C5aR, albeit at lower potencies than intact C5a. This C-terminus is proposed to act at the second extracellular loop of C5aR [5].

Given the proposed involvement of C5a in classical inflammatory diseases such as rheumatoid arthritis, [6] and a widening of the pathogenic roles of C5a to traditionally, non-inflammatory diseases such as cancer [7], and brain diseases [8], there has been a burgeoning interest in developing inhibitors of C5a for clinical use [9]. One such method has been to block C5a-C5aR interaction through the development of selective C5aR antagonists. In 1999 and 2000, a series of cyclic peptide full antagonists were described which were designed around the C-terminal residues of C5a [10,11]. One of these compounds, termed 3D53 (later named PMX53), has been the most extensively studied, and is widely used to study C5aR function in disease [3]. It is a cyclic hexapeptide (structure: Ace-Phe-[Orn-Pro-dCha-Trp-Arg]), which has low nanomolar potency, and efficacy in a wide range of species [12], making it an ideal research tool [3]. One potential drawback for the clinical use of this compound, however, is its peptidic nature and low oral bioavailability [13], which has limited its commercial development [9].

The recent determination of several GPCR crystal structures has contributed significantly to our understanding of GPCR structural organization, ligand specificity, and activation [14-19]. MD simulations have provided considerable insight on the conformations of inactive states and the mechanism of receptor activation (Ref. [20] and references therein). Nevertheless, the generation of reliable structural models for GPCR proteins of unknown structure and for their ligand complexes remains a challenging task [21].

In the present study we develop molecular models of PMX53 bound to C5aR in a model membrane. Our goal is to use the resulting models to develop peptidomimetics [22] and chemical compounds, which may overcome some of the clinical hurdles that were encountered with PMX53 [3]. We start from a well-tested earlier structural model for free C5aR [23-27] and an NMR structure for PMX53 [28], and generate a large number of representative conformations for free C5aR and the PMX53 ligand by multi-ns MD simulations. Docking of the obtained conformations yields a large number of structural models for the complex (~300,000 structures); subsequent filtering by structural and energetic criteria identifies a manageable number of candidate complexes, which are further tested by extensive MD simulations and free energy calculations. In contrast to previous computational studies of the C5aR complexes with C5 and PMX53, we represent membrane effects on the protein and ligand interactions at the MD simulation and assessment stage via high-quality implicit-membrane models [21,29-31]. Such models offer a promising alternative to explicit-membrane treatments, which are more accurate but are impractical for structure-prediction calculations [32,33]. Furthermore, the implicit-membrane representation enables the rapid determination of binding free energies for several hundred thousand candidate structures. Even though no experimental information has been incorporated *a priori* in the docking, the most promising complexes are consistent with available experimental data, reflecting the accuracy and potential of the employed methodology.

The obtained models for the C5aR:PMX53 complex can serve as the basis for knowledge-based discovery of C5aR antagonists with improved properties compared to PMX53, as well as for basic mechanistic studies of complement activation and function at molecular detail and atomic resolution. Furthermore, the described combination of implicit-membrane MD simulations, docking and free energy calculations is a promising framework for the generation and assessment of structural models for GPCR-ligand complexes.

## Methods

### Description of simulation systems

#### C5aR receptor

The human GPCR receptor C5aR consists of 350 amino acids, and has the typical GPCR topology, with an extracellular N-terminal fragment, seven trans-membrane (TM) helices interconnected by extracellular (EC) and intracellular (IC) loops, and an intracellular C-terminal fragment [26]. Nikiforovich et al. has constructed structural models for free C5aR [24] as well as its complex with C5a [25,26]. In the MD simulations we use as a starting point for C5aR the structural model of Nikiforovich [24-26]. The seven transmembrane helices in the Nikiforovich model are defined as: 38–63 (H1), 71–98 (H2), 107–138 (H3), 150–172 (H4), 199–224 (H5), 236–267 (H6), and 281–300 (H7); similarly, the three extracellular loops are defined as 99–106 (EC1), 173–198 (EC2), and 268–280 (EC3) [26]. The simulation system omits the first seven amino acids, which do not contribute to C5a binding and are not expected to affect binding of PMX53. It also omits the intracellular C-terminal region 307–350, which is very remote from the insertion point of the C5a C-terminal end, and the putative ligand binding site.

#### PMX53 ligand

**Figure 1:**
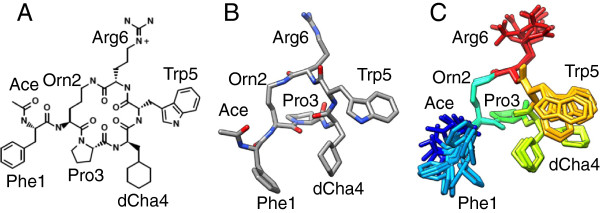
**Structure of the cyclic hexapeptide ligand PMX53, with amino acid sequence Ace-Phe-[Orn-Pro-dCha-Trp-Arg]. Panel A**: two-dimensional Chemical structure PMX53. The N-terminal end is blocked by the group CH3-CO (Ace); Orn denotes ornithine and dCha d-cyclohexyl-alanine. The brackets denote cyclization via a covalent bond between the Orn side-chain and the Arg6 carbonyl group. **Panel B**: three-dimensional representation of the first conformer of the NMR ensemble of structures of PMX53. Atoms are colored according to element type. **Panel C**: three-dimensional representation of the ensemble of the NMR structures of PMX53. The color of residues transitions from blue to red between the N- and C- termini. Hydrogens are omitted for clarity.

The hexapeptide PMX53 (Figure 1) has the sequence Ace-Phe-[Orn-Pro-dCha-Trp-Arg]; Ace denotes the blocking group CH_3_-CO at the N-terminal end, Orn ornithine, dCha d-cyclohexyl-alanine, and the brackets denote cyclization of the mainchain via a covalent bond between the Orn side-chain and the Arg6 carbonyl group. Figure 1A shows the chemical structure of PMX53, and Figure 1B,C shows three-dimensional representations of the NMR structure of PMX53 [28].

### Construction of structural models for the C5aR:PMX53 complex

In order to achieve a systematic construction and evaluation of plausible structural models for the complex, we employed a range of methods, including MD simulations, docking, energy minimizations, and binding-affinity calculations. Our computational framework consisted of the following steps: (A) generation of a large collection of representative PMX53 and C5aR structures via long MD simulations of the isolated ligand and receptor; (B) clustering of the simulation trajectories and determination of high-probability conformations; (C) generation of a large number of potential structural models for the complex, via docking of conformations from the most populated PMX53 and C5aR clusters; (D) filtering of the models with structural and energetic criteria; (E) assessment of the most promising models by MD simulations and binding free energy calculations. Each step is detailed below.

#### Generation of PMX53 conformations

Competition binding studies with linear and cyclic peptide antagonists suggest that the binding site of PMX53 is in the transmembrane region of C5aR, near or at the location of the binding site of the C5a C-terminal end [5]. NMR experiments [28] have shown that the dominant conformation of *free* PMX53 in deuterated DMSO (DMSO-d6) has residues 1–2 in a random-coil state, and segment 3–6 in a type-II β-turn. PMX53 may assume a different conformation in the complex with C5aR, due to the influence of the surrounding C5aR receptor and the embedding membrane. Hence, it is important to generate alternative structural models of PMX53, which are likely to be stabilized by the less polar environment of the binding site. As an approximation, we generated such conformations by MD simulations of free PMX53 in a membrane environment, represented by the implicit-membrane molecular-volume Generalized Born (GBMV) model [31]. Standard amino acids were described by the CHARMM all-atom topology and energy function [34], using the CMAP correction for all L-amino acids [35]. Topology and parameters for non-standard groups (ornixthine, cyclohexylalanine and the Orn2-Arg6 cyclization segment of PMX53) were derived, respectively, from CHARMM22 definitions for lysine, alanine/cyclohexane and the peptide group.

To represent conditions of variable polarity, we conducted four simulations, in which we restrained the PMX53 center of mass at 0 Å, 10 Å, 15 Å and 20 Å, respectively, from the membrane center. The total simulation length was 180 ns (45 ns per position). We also conducted an additional 15-ns run in aqueous solution, represented by the corresponding aqueous GBMV model [30]. All simulations were conducted with the CHARMM program, version c35b3 [36].

#### Classification of PMX53 conformations

The dominant simulation conformation of the PMX53 mainchain was in agreement with the NMR structure [28], with residues 1–2 in random coil and segment 3–6 in a type-II β-turn. We partitioned the conformations into groups, based on the following nine side-chain dihedral angles: (i) 1N-1C_α_-1C_β_-1C_γ_, (ii) 1C_α_-1C_β_-1C_γ_-1C_δ1_, (iii) 4N-4C_α_-4C_β_-4C_γ_, (iv) 4C_α_-4C_β_-4C_γ_-4C_δ1_, (v) 5N-5C_α_-5C_β_-5C_γ_, (vi) 5C_α_ -5C_β_-5C_γ_-5C_δ1_, (vii) 6N-6C_α_-6C_β_-6C_γ_, (viii) 6C_α_-6C_β_-6C_γ_-6C_δ_, (ix) 6C_β_-6C_γ_-6C_δ_-6N_ϵ_. We employed the clustering algorithm of the CHARMM program [37,38], a clustering radius of 45° and a “maximum error” radius (reflecting the maximum difference among the centers of distinct clusters) of 10°. We obtained a total of 775 clusters, with 51 clusters containing ca. 30% of the conformations. We employed representative structures from all 51 clusters in the docking calculations. The clustering focused on side-chain dihedral angles as the side-chains are significantly more flexible than the backbone. For the 51 clusters, the average root-mean-square difference (RMSD) of the backbone/side-chain heavy atoms with regard to the average structure of the 51 clusters is equal to 0.54 ± 0.15 Å/2.46 ± 0.52 Å. A significantly higher mobility of the side-chains compared to the backbone is also present in the 10 NMR-derived structures (see Figure 1C), according to which the average RMSD of the backbone/side-chain heavy atoms with regard to the average NMR structure of the 10 conformations is equal to 0.23 ± 0.07 Å/1.85 ± 0.22 Å [28].

#### Generation of C5aR conformations

The initial C5aR conformation was taken from the Nikiforovich model for the C5aR:C5a complex [25]. Details on the construction of the structural models for C5aR and the C5aR:C5a complex can be found in previous work [25,39].

Starting from this structure, we generated conformations of C5aR via MD simulations with the implicit-membrane switching-function generalized Born (GBSW) module [29]. Two parameters of this model are the total thickness of the low-dielectric membrane slab (*T*_*memb*_) and the half-length (*l*_*msw*_) of the membrane/water interface buffer region. Based on UNIPROT [40] definitions, we estimated the total thickness of the intra-membrane C5aR helical region at ~ 35.5 Å. At the same time, the OPM database [41] yields a hydrophobic thickness of 32.2 ± 1.2 Å for rhodopsin (PDB code 1GZM), the protein on which the C5aR model is based. We combined the OPM and UNIPROT predictions, by setting *T*_*memb*_ to 36 Å and *l*_*msw*_ to 2.5 Å. With these values, the combination *T*_*memb*_ – 2* *l*_*msw*_ is 31 Å, i.e. corresponds to the lower bound of the OPM estimate. Test simulations indicated a stable behavior (RMSD of ~3.0 Å from the initial conformation), whereas somewhat different *T*_*memb*_ values resulted in larger RMSD from the initial conformation (~3.5 Å). A similar membrane thickness (35 Å) and half-length (2.5 Å) were used in recent structural modeling of class A GPCRs, which employed the same implicit-membrane model [21]. The surface tension coefficient (γ) was set to 0.04 kcal/(mol Å^2^); other parameters were set to default values of the GBSW model [21].

To enlarge the ensemble of generated C5aR conformations, we conducted simulations with four protocols: In protocol (i) we removed the C5a ligand and simulated the C5aR protein at an elevated temperature (400 K), with harmonic restraints on the heavy backbone atoms of the entire protein, and the side-chains unrestrained. In this way, the C5aR binding pocket retained a similar volume as in the C5aR:C5a complex (Nikiforovich model) [25], while the simulation eliminated any bias in the initial side-chain conformations, which might arise due to interactions with the C5a ligand. In protocols ii-iii we simulated the complex between C5aR and the C5a fragment 60–74, which binds at the intra-membrane C5aR region. The C5a fragment was either restrained near its initial shape via the *bestfit* option of CHARMM (protocol ii), or was left unrestrained (protocol iii). Finally, in protocol iv we simulated the entire C5aR:C5a complex without restraints. The simulation temperature of protocols ii-iv was 300 K. Prior to each production run, we subjected each system to energy-minimization, heating, and 1.6-ns equilibration. The length of the production run was 11 ns for protocol iii, and 5.5 ns for all other protocols.

Standard amino acids were described by the CHARMM all-atom topology and energy function [34] including a CMAP correction of the backbone torsional angle energetics [35]. A 16-Å cutoff distance was used for non-bonded interactions. The lengths of covalent bonds containing hydrogen atoms were constrained by the SHAKE algorithm [42], and the equations of motion were solved with an integration time step of 2.0 fs. The system was in contact with a Langevin heat bath at 300 K; a friction coefficient of 5 ps^−1^ was used for heavy atoms.

#### Classification of C5aR conformations

We characterized the C5aR conformations by the shape of their intra-membrane binding pocket, since structural differences in other protein regions should not be as relevant to PMX53 binding. We described the shape of the binding pocket by a novel methodology, which filled the binding cavity (after removal of C5a) with particles having the approximate diameter of a water molecule and taken from a water box; these particles created a grid of points, with an inter-point spacing equal to the water diameter. The underlying premise was that by ensuring that the particles were always in the same position relative to C5aR, it would be possible to identify the cavity regions that were changing structurally, by simply keeping track of which particles fit in the cavity. To create an ensemble of cavity shapes, we first superimposed each MD snapshot onto the initial structure (the Nikiforovich model), based on the Cα-atoms of the C5aR trans-membrane regions; we then overlayed a 50 Å × 50 Å × 60 Å explicit water box on each snapshot, and deleted all water molecules whose oxygen atom was within 2.4 Å of any C5aR heavy atom. Even though C5a was not taken into account when computing the cavity shapes, water molecules within 4 Å of the coordinates of the 15 C-terminal residues (60–74) of C5a were used to define the top of the cavity; water molecules further than 4 Å from these residues were removed.

We created lists identifying the remaining molecules, and used them to generate binary water fingerprints with length equal to the number of unique water molecules found in the cavities of the MD snapshots. A value of 1 (0) was associated with present (absent) water molecules. We quantified the similarity between two water fingerprints A and B via distance matrices based on the Jaccard binary distance measure [43].

(1) $$J_{\mathrm{AB}}=\frac{C_{10}+C_{01}}{C_{10}+C_{01}+C_{11}}$$

In Eq. (1), *C*_*11*_ is the number of common water molecules in both fingerprints (cavities), and *C*_*10*_, *C*_*01*_ are, respectively, the numbers of water molecules only in fingerprint A or B. The Jaccard distance varies from 0 (fingerprints with the same water molecules) to 1 (no water molecules in common). We then used hierarchical clustering [44] to classify the MD snapshots into families, based on water fingerprint similarity, and visualized the clustering via dendrogram trees. The dendrogram trees were cut at an arbitrary binary similarity value of 0.3, to keep the number of selected structures manageable. With this value, two structures not in the same cluster differed by more than 50 water molecules. From each cluster, we selected as most representative the cavity structure with the largest volume (largest number of present water molecules). Ultimately, we chose 150 – 250 structures from each protocoll (a total of 785 structures) for the docking studies. These calculations were performed with scripts written in the R statistical language [45] using the Bio3D [46] library. The above methodology was not employed to the high-temperature simulation (protocol i), which was restrained to maintain the volume of the binding cavity; from that trajectory, we simply extracted structures at intervals of 40 ps.

#### Docking

Using representative C5aR and PMX53 snapshots from the structural classification described above, we generated possible structures of the C5aR:PMX53 complex with the program DOCK6 [47]. For each C5aR snapshot, we created the corresponding molecular surface via DMS [48], and represented potential binding sites by spheres via the SPHGEN utility. SPHGEN identifies spheres tangent to the molecular surface, and performs clustering to eliminate redundant spheres, which represent surface cavities and serve as potential locations of atoms during ligand orientation. For each C5aR snapshot combination, we only retained spheres within 7 Å of the coordinates for the 15 C-terminal residues of C5a. For protocols ii-iv, the coordinates for C5a were taken from the specific snapshot; for the elevated temperature run, the C5a coordinates were taken from the Nikiforovich model. A receptor-ligand clash was defined as >50% atom-atom overlap of two atomic radii; we excluded docking poses with more than five such clashes. All orientations passing this filter were saved as input for the CHARMM-based scoring procedure, since the docking program was not used to rank the C5aR:PMX53 poses, due to the complexities of GPCR docking.

Altogether, 51 PMX53 conformations, corresponding to the most populated 51 clusters from the PMX53 trajectory analysis, were docked to the representative structures from each of the four C5aR MD simulations, resulting in 306,497 structural models for the complex. A rigid docking procedure was used; nevertheless, the use of flexible templates of the ligand and the receptor incorporate flexibility in the docking.

#### Filtering of docked structures

**Figure 2:**
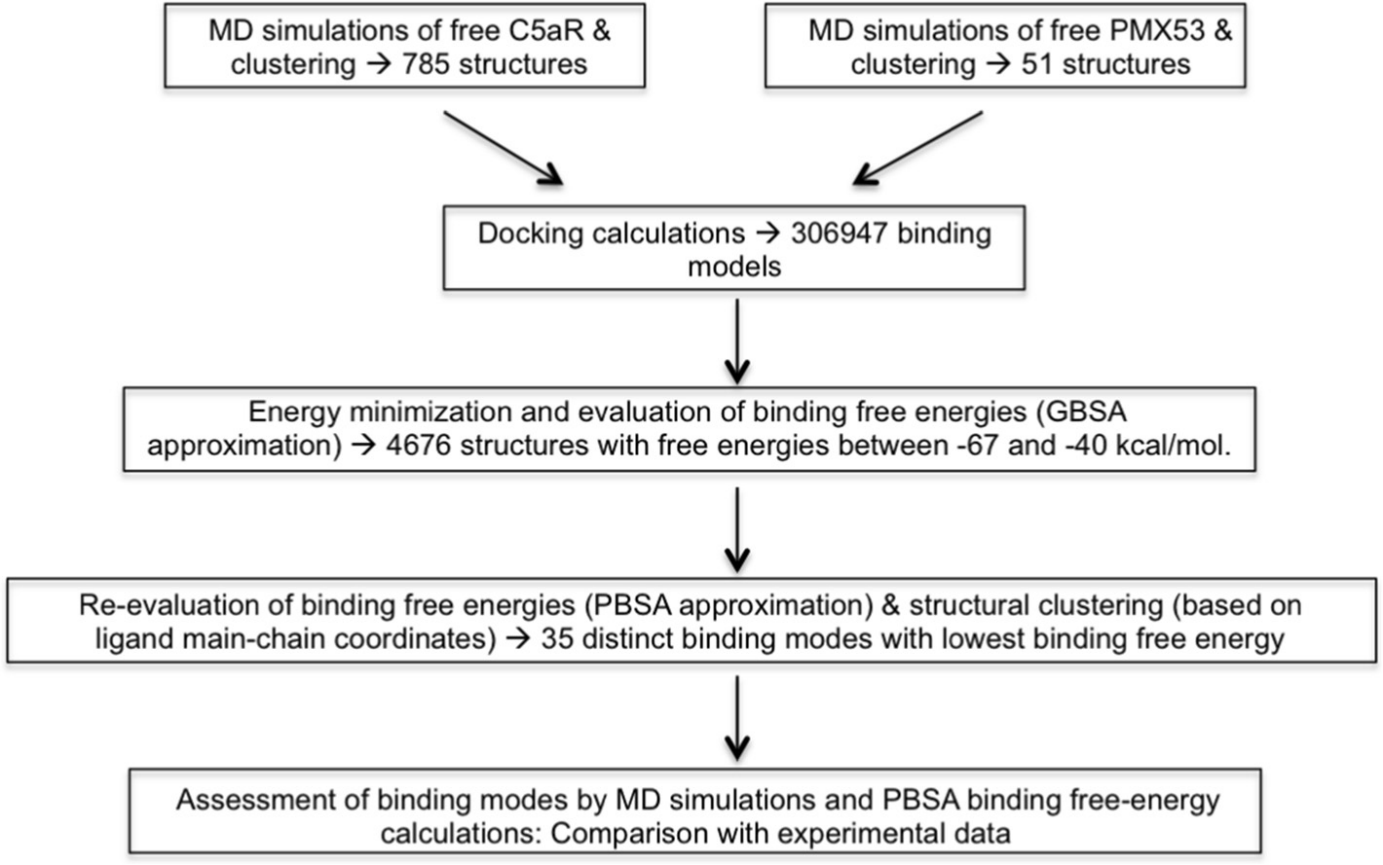
Flowchart of the computational framework used to generate and assess structural models of the C5aR:PMX53 complex.

We subjected all 306,497 docking conformations to 100 energy steepest-descent minimization steps in a uniform dielectric medium (ϵ = 4), followed by 50 steepest-descent minimization steps in the GBSW implicit-membrane environment. At the end of minimization we filtered the conformations by a combination of energy-based and structure-based criteria. A flowchart is presented in Figure 2.

The energy-based criterion was applied in two steps: First, we computed the binding free energies of all 306,497 conformations (at the end of minimization) in the Molecular Mechanics Generalized Born Surface Area Approximation (MM-GBSA) [49-51], via the following equation

(2) $$\mathrm{\Delta G}=G_{\mathrm{PL}}-G_{P}-G_{L}$$

In Eq. (2), *G*_X_ is the total free energy of molecule X (complex PL, free protein P, or free ligand L). The protein and ligand conformations were assumed identical in the complex and free molecules. With this assumption, any bonded-energy contributions to ΔG cancel in Eq. (2). Even though this assumption is not strictly accurate, it is probably sufficient for the identification and rejection of conformations with weak association free energies. Protein, ligand and complex entropic contributions are ignored; they are expected to approximately cancel when comparing relative affinities of different binding modes. The solvation components of the complex and free protein free energies were computed in the inhomogeneous membrane/water environment; the solvation component of the unbound ligand free energy was computed in aqueous solution (modeled by the implicit GBSW model) [29]. We thus identified 4676 structures (resulting from the minimization of the 306,497 docking conformations), whose binding free energies were within 27 kcal/mol of the binding free energy minimum (−67 kcal/mol).

In the second filtering step with the energy-based criterion, we calculated the binding free energies of these 4676 structures in the Molecular Mechanics Poisson-Boltzmann Surface Area Approximation (MM-PBSA) [49]. In these calculations, we inserted the complex and protein in a membrane slab with a thickness of 31 Å and a dielectric constant of 1, surrounded by water with a dielectric of 80. The protein dielectric constant was set to 2. The free ligand was placed in pure water. The MM-PBSA calculations were performed with the Poisson-Boltzmann solver of the CHARMM program (PBEQ module). We used 150 grid points in each direction and a grid-spacing of 0.5 Å. The MM-PBSA calculation yielded a more expanded free energy range (between −90 kcal/mol and −20 kcal/mol), and changed the relative affinity for some structures. The correlation between MM-GBSA and MM-PBSA was analyzed via a linear least squares fit; the slope and the standard error, respectively, were equal to 1.03 and 0.03.

In the next step, we used a structure-based criterion in conjunction with the MM-PBSA binding free energies, to identify structurally distinct, high-affinity binding modes. Clustering analysis of the PMX53 mainchain coordinates (N, CA and C atoms) with a 5-Å clustering radius identified 35 clusters, each reflecting a distinct ligand-binding mode. The analysis was performed with the program WORDOM [52]. From each cluster we extracted the conformation of lowest MM-PBSA binding free energy, and used it as a starting point in MD simulations.

### Evaluation of structural models for the C5aR:PMX53 complex by MD simulations

Prior to the MD runs, we relaxed each structure via 1000 steps of steepest-descent energy minimization. Subsequently, we heated the complexes to a temperature of 300 K via four 100-ps runs with respective temperatures of 75 K, 150 K, 225 K and 300 K; during the heating stage we restrained all heavy backbone atoms to their initial positions by a harmonic force with strength of 5 kcal/mol/Å^2^. We then conducted three 400-ps equilibration runs at 300 K, in which we gradually reduced the harmonic-restraint force constant to 1 kcal/mol/Å^2^ for intra-membrane residues, and to zero for residues in extra-cellular (EC) and intra-cellular (IC) loops. In the subsequent production runs, we removed all protein and ligand restraints. Parameters and simulation details were the same as for the free ligand and C5aR simulations, described above. The duration of each production run was 7 ns, with only the last 4 ns employed in the analysis.

In order to assess the stabilities of the simulated complexes, we computed the corresponding MM-PBSA association free energies. For each run, the results were averaged over 700 snapshots extracted at 10-ps intervals. The best complex had a free energy of −175 kcal/mol. Seven complexes within 20 kcal/mol of the lowest value (−175 kcal/mol) of less than −155 kcal/mol (20 kcal/mol greater than the average of the most promising complex) were simulated for an additional 13 ns (a total of 20 ns); the last 17 ns were employed in the analysis.

### Residue pairwise interaction free energies

We analyzed the intermolecular interaction free energies between PMX53 and C5aR residue pairs of the final simulation using the following equation:

(3) ΔGRR'inte=∑i∈R∑j∈R'EijCoul+GijGB︸ΔGRR'polar+∑i∈R∑j∈R'EijvdW+σ∑i∈R,R'ΔSi︸ΔGRR'nonpolar

The first and second group of terms on the right-hand side of Eq. (3) describe, respectively, polar and non-polar interactions between a C5aR residue R and a PMX53 residue R’. The polar component contains a Coulombic energy term, and a free energy GB contribution, modeling the interaction between residue R and the solvent polarization potential induced by R’. Similarly, the non-polar component contains a van der Waals interaction between R, R’ and a surface free energy term, expressing cavity contributions and nonpolar interactions with the surrounding solvent.

The non-polar and polar solvation terms were calculated using the implicit membrane GBSW. The generalized-Born energies and the atomic accessible-surface areas (ΔS_i_) entering in Eq. (3) depend on the location of R and R’ in the complex. To compute the polar (GB) interaction free energy term for a particular residue pair (R, R’) in Eq. (3), we set to zero the charges of atoms not in residues R and R’. The surface term contains the difference in solvent accessible surface areas of residues R and R’ in the complex and unbound states; the interaction free energy non-polar term represents the creation of a cavity in its surrounding medium (membrane/water) to accommodate biomolecules and switching-on dispersion interactions between biomolecules and the surrounding medium while all atomic charges are set to zero [51]. A similar methodology for the analysis of interacting residues has been used for the elucidation of species-specificity of complement protein C3 [53] and the design of transgenic (mouse/human) C3 with putative affinity for compstatin [54].

## Results and Discussion

### Earlier models of free C5aR, and the complex of C5aR with its natural activator, peptide C5a

The C5aR protein has the typical GPCR topology, with an extracellular N-terminal fragment, seven trans-membrane (TM) helices interconnected by extracellular (EC) and intracellular (IC) loops, and an intracellular C-terminal fragment [55]. Nikiforovich et al. has constructed structural models for free C5aR [24], as well as its complex with C5a [25,26]. The structure of the C5aR TM region in these models was based on the corresponding TM region of dark-adapted rhodopsin, and the loops were constructed with a *de novo* structure prediction method [23]. The model has been used to interpret experimental results on C5a binding to C5aR [25], and to model the conformational changes occurring during receptor activation in the TM region [56] and EC loops [27] of C5aR. The loop prediction method was also applied with success to construct structural models of extracellular loops for other GPCR receptors [57].

Numerous systematic experimental studies have led to a two-site model of C5aR activation (see [2,3] and references therein). The primary affinity site involves contacts between acidic residues in the N-terminal end of C5aR and basic residues in the core of C5a; interactions in this site contribute to binding strength, but not to C5aR activation. The second site is formed by residues in the TM domain and the EC loops and interacts with the C5a terminal fragment 60–74. This site contributes to the activation of C5aR by C5a and other peptide agonists.

Experiments with agonist and antagonist peptide mimics and receptors mutated at possible interaction sites have provided information on the ligand binding site of C5aR. Competition-binding experiments suggested that PMX53 binds at or near the same TM location as the C-terminal moiety of C5a [5]. The substitution Ile116Ala correlated with the emergence of weak agonist activity in a PMX53 derivative with a bulkier side-chain (benzothiazolalanine) at position 5 [5], and converted a linear peptide mimic from antagonist to agonist [58]. These results suggested that residue 5 of PMX53 (and related peptides) binds in the vicinity of Ile116. The C5aR substitution Asp282Ala (in the EC3 loop) caused a 10-fold reduction in PMX53 affinity, suggesting the formation of an electrostatic interaction between Arg6 of PMX53 and Asp282. The substitution Arg175Asp converted PMX53 to a weak agonist, indicating that Arg175 might play a role in the discrimination between agonists and antagonists; additional experiments suggested a possible interaction between Arg175 and the C-terminal carboxylate of hexapeptide ligands [5]. Mutations Arg206Ala and Glu199Lys also affected the receptor activation by hexapeptide ligands; modeling in [5] argued that a possible interaction Arg206-Glu199 may stabilize the position of helix H5, and disruption of this interaction may contribute to activation.

Taking into account these experimental results, Higginbottom *et al*. constructed two models for the PMX53:C5aR complex [5]. The C5aR conformation was based on homology with dark-adapted rhodopsin. Loop EC2 was kept in a “closed” position (near the membrane), via a Cys109-Cys188 disulfide bond. Various docking conformations of PMX53 inside the C5aR binding pocket were tried, under the condition that they satisfied two ligand-receptor interactions: (i) The center of the Trp5 indole ring was restrained within a distance of 5 Å from the Cα atom of Ile116, and (ii) the side-chain of Arg6 formed a salt-bridge with the side-chain of Asp282. In the resulting model, the PMX53 Phe1 side-chain was positioned between helices 6 and 7, in the vicinity of residues Ile263 (H6) and Leu207, Phe211 (H7); the nonpolar dCha4 side-chain was directed toward helices 3 and 4, near Pro113, Ser114, Arg197 and the Glu199-Arg206 salt bridge; Trp5 was placed near the hydrophobic residues Pro113, Ile116 and Val286; finally, Arg6 was proximal to Asp191, Tyr258 and Gln259, in addition to its salt-bridge with Asp282. The final model was presented in Figure five(B) of [5]. Higginbottom *et al*. [5] argued that the activation of C5aR may be prompted by the insertion of the Arg6 side-chain at a buried position in the ligand binding pocket, past Ile116. On the other hand, the Ar6-Asp282 interaction in the modeled C5aR:PMX53 complex prevents the deep insertion of the Arg6 guanidinium group into the pocket, enabling the antagonist activity of PMX53. A second model resulted from docking attempts of PMX53 onto C5aR with the same two constraints (Trp5-Ile116, Arg6-Asp282), but with the disulfide bridge Cys109-Cys188 absent and the C5aR EC2 loop in an open conformation. In the resulting model, (Figure five(C) of Higginbottom *et al.*) [5], the ligand was positioned with the Phe1 side-chain at a similar position (between helices H6 and H7), but with the Arg6 and dCha4 interactions swapped. We note that the covalent disulfide bridge Cys109-Cys188 should also be present in the open EC2 conformation. As we show below, PMX53 is inserted in the second recognition region, near the modeled location of the C5a C-terminal segment 69–74.

### Ranking of the most promising C5aR:PMX53 complexes

A detailed description of our modeling procedure is presented in the *Methods*, and a summarizing flowchart is shown in Figure 2. We first generated a large number of representative conformations for the ligand and the receptor by MD simulations, and grouped them into conformational clusters. We then performed docking calculations of protein and ligand conformations, without any geometrical restraints. Using a filtering calculation, consisting of conformational clustering, energy minimization and binding free energy evaluations, we identified 35 promising binding modes covering the entire TM cavity of C5aR. The MM-PBSA binding-free energies of these modes ranged between −90 and −65 kcal/mol (Additional file 1: Figure S1).

We assessed further the stabilities of all 35 structural modes by implicit-membrane MD simulations of the C5aR:PMX53 complex. In all cases, the initial conformations were well preserved; the average RMSD of all TM backbone protein residues was 3.3 ± 0.2 Å relative to the structure of Nikiforovich *et al*. [25] MM-PBSA analysis of the simulation trajectories (up to 7 ns) showed that seven structural modes (22, 6, 3, 30, 1, 4, and 10) had significantly lower binding free energies. The simulations of these models were extended to 20 ns; this length was sufficient for convergence of the MM-PBSA association free energies (not shown).

The free energy values (averaged over the last 17 ns for the 20-ns trajectories, or last 4 ns for the 7-ns trajectories) are plotted in Additional file 1: Figure S1 as red diamonds. The averaged-over-simulation MM-PBSA affinities are lower than the initial values of the same models. This is mainly due to the vdW component, reflecting the improvement of protein-ligand contacts with respect to the initial docked structures.

Structural mode 22 has the lowest association free energy (−175 kcal/mol). Interestingly, this mode agrees with experimental data and with features of an earlier mode proposed by Higginbottom *et al*. [5] The next best modes (6 and 3) have association free energies of −166 kcal/mol, ~10 kcal/mol higher relative to mode 22. Mode 30 has a comparable association free energy with 6 and 3 (−165 kcal/mol), but involves an entirely different ligand orientation. All four modes are described below.

### Structural analysis of mode 22 and comparison with experimental data

**Figure 3:**
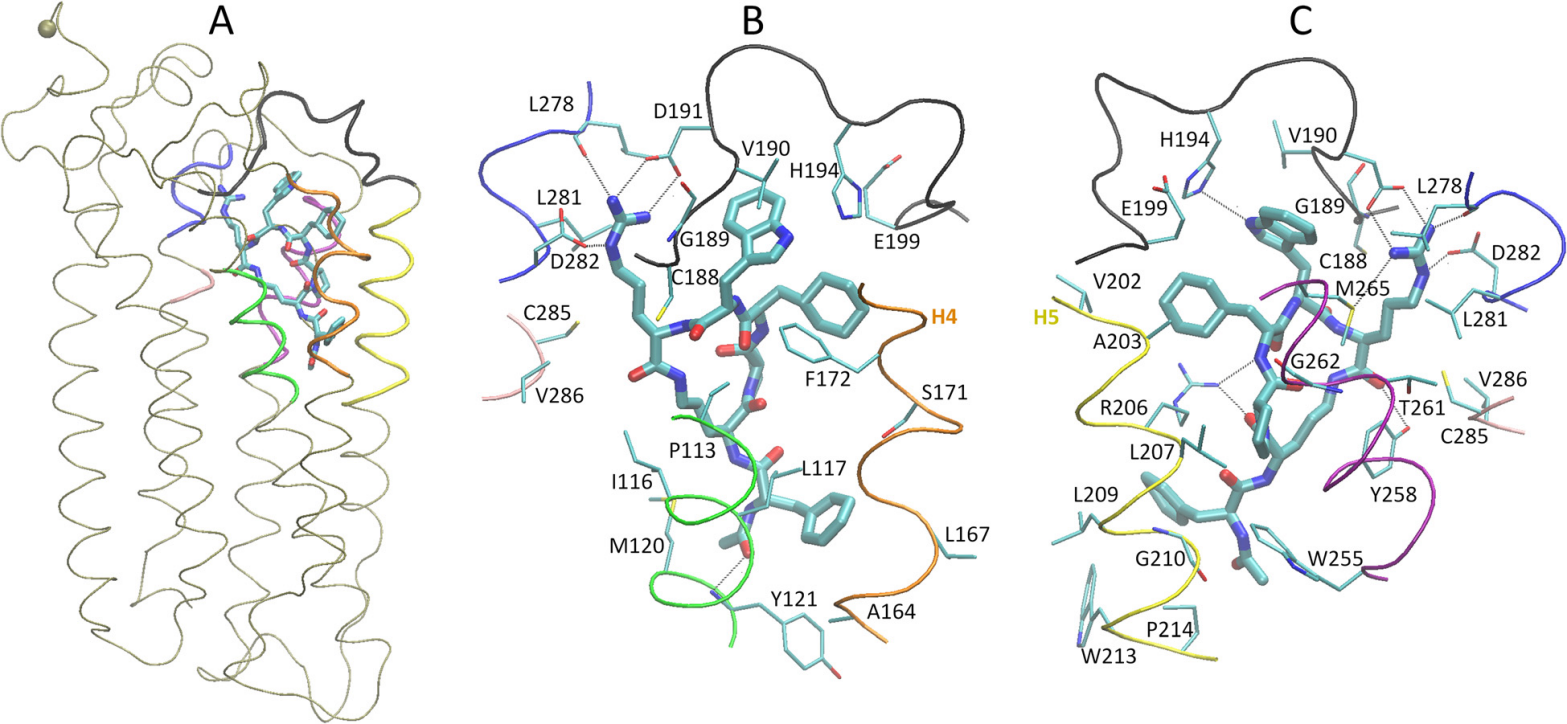
**The structure of binding mode 22. Panel A**: The C5aR backbone is shown as a thin tan tube. The first 7 residues are omitted as in the Nikiforovich model [25]; the Cα atom of the 8^th^ residue is depicted as a vdW sphere. Protein segments in contact with PMX53 are indicated in the following colors: 113–121 (H3) in green, 164–172 (H4) in orange, 188–199 (EC2) in black, 202–214 (H5) in yellow, 255–265 (H6) in purple, 278–282 (EC3) in blue, and 285–286 (H7) in pink. **Panels B** and **C** present a close-up view of the binding mode and interactions. Panel **C** is rotated by 180° around a vertical axis, with respect to panel **B**. Domains H6 and EC3 are omitted from panel **B**, and domains H3 and H4 are omitted from panel **C**, for clarity. H4 and H5 are additionally denoted in panels **B** and **C** to assist the reader. In panels **B** and **C**, PMX53 atoms are shown in thick licorice; selected C5aR mainchain and side-chain heavy atoms interacting with PMX53 are shown in thin licorice. All atoms are colored by atom type. Hydrogen-bond interactions are shown as black, dashed lines.

The conformation of mode 22 is displayed in Figure 3. PMX53 is surrounded by helix 3, helix 4, the extracellular domain EC2, helix 5, helix 6, the extracellular domain EC3, and helix 7. The N-terminal end of the ligand points toward the membrane interior, with the center-of-mass (COM) of the Ac-Phe1 mainchain at a distance of ~ 5 Å from the membrane center. The C-terminal end of the ligand is directed toward the membrane-water interface; the COM of the Arg6 mainchain lies at the interface, at a distance of ca. 14 Å from the membrane center (Figure 3A).

**Figure 4:**
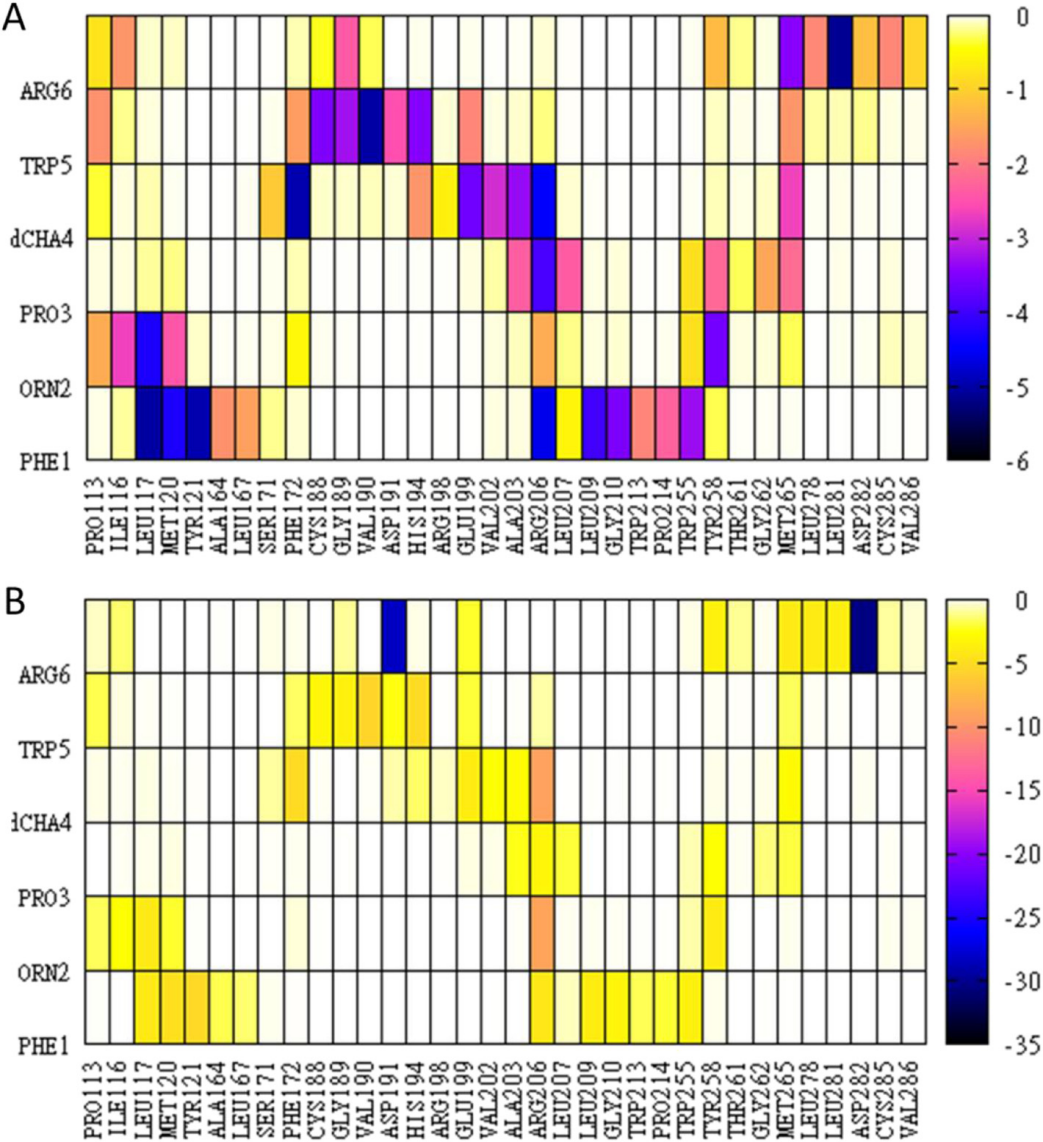
**MM-GBSA interaction free-energies (kcal/mol) for all interacting protein-ligand residue pairs in binding mode 22. Panels A** and **B** correspond, respectively, to energies of non-polar and polar interactions, as defined in Eq. (3).

**Figure 5:**
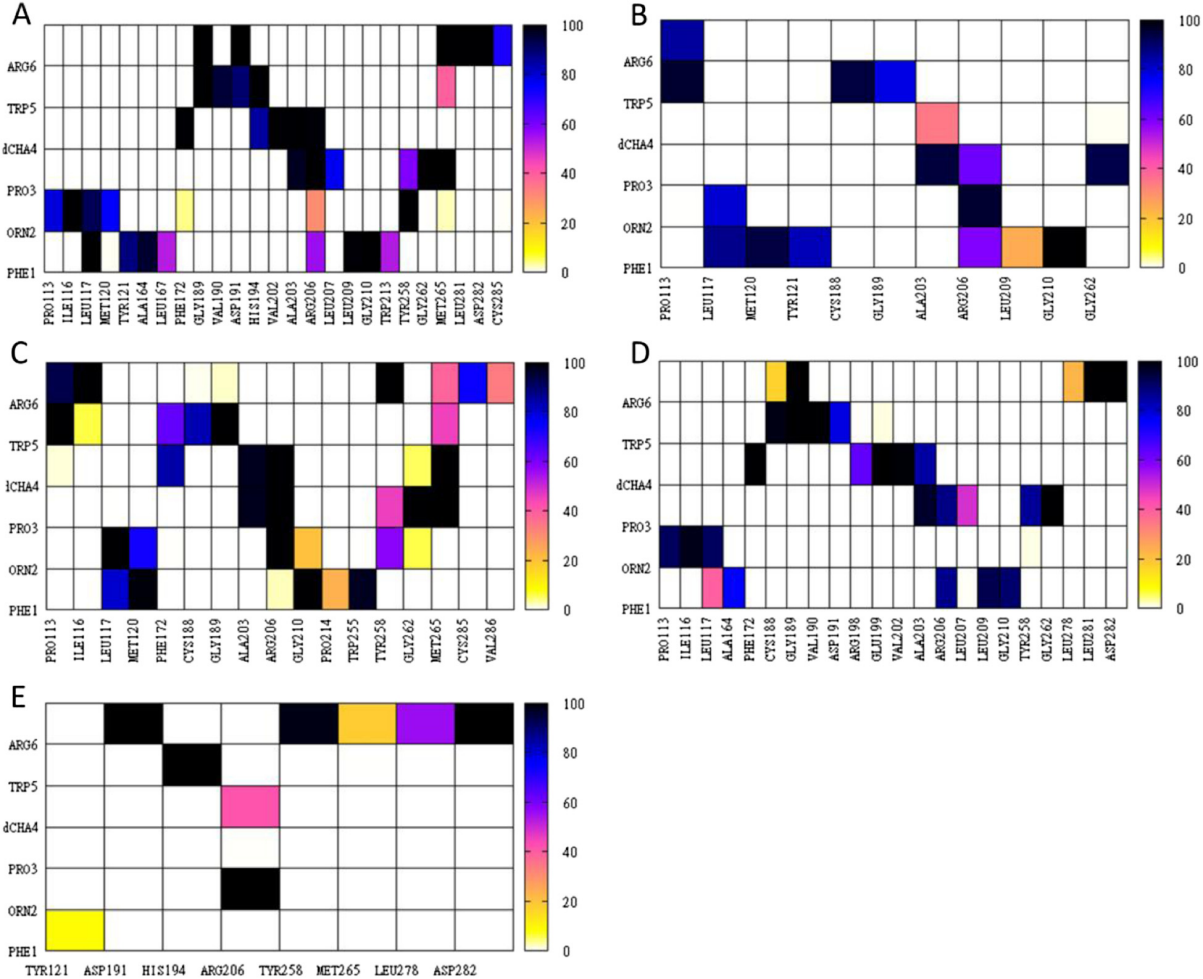
**Probability (%) maps of C5aR:PMX53 intermolecular contacts and hydrogen bonds, computed from the MD simulation trajectory of binding mode 22. Panels A**, **B**, **C** and **D**, show, respectively, protein side-chain – ligand side-chain, protein mainchain – ligand mainchain, protein side-chain – ligand mainchain, and protein mainchain – ligand side-chain contacts. A contact was considered present in a trajectory snapshot, if the distance betweeen the geometric center of the corresponding moieties was smaller than 6.5 Å. Panel **E** displays a probability map (%) of C5aR:PMX53 hydrogen bonds. A hydrogen bond among two heavy atoms was considered present if the donor-acceptor distance was smaller than 3.5 Å and the D-H – A angle was larger than 90°.

A close-up view of the complex conformation in the vicinity of PMX53 is shown in Figure 3 (panels B and C). Interaction free energy components of all protein-ligand residue pairs are displayed in Figure 4, and contact maps are shown in Figure 5. Furthermore, a list of PMX53-C5aR residue pairs with total intermolecular residue-pair energies of at least 1.8 kcal/mol, as evaluated by Eq. (3), is included in Table 1.

**Table 1:** Residue pairs forming strong intermolecular interactions in binding mode 22

**PMX53**	**C5aR**^**1**^
Ac-Phe1	Tyr121, Met120, Arg206, Leu117, Leu209, Trp255, Gly210, Pro214, Ala164, Trp213, Leu167
Orn2	*Arg206*, Leu117, Tyr258, Ile116, Met120, Pro113
Pro3	Arg206, Tyr258, Ala203, Leu207, Met265
dCha4	*Arg206*, Phe172, Glu199, Met265, Ala203, Val202, His194
Trp5	Val190, *His194*, Gly189, Cys188, Asp191, Glu199, Pro113, Met265, Phe172
Arg6	*Asp282*, *Asp191*, Met265, *Leu278*, Leu281, *Tyr258*, Ile116

^1^Intermolecular energies were computed with Eq. (3) and were averaged over the simulation trajectory; only pairs with total energies of at least 1.5 kcal/mol are tabulated. In each line, the C5aR residues are listed in descending order of total interaction energy with the corresponding PMX53 residue. Italics denote C5aR residues engaged in strong polar interactions.

Ac-Phe1 is inserted into a binding pocket formed by helices H3, H4, H5 and H6. The acetylated N-terminal end is positioned in the interior of a triangle formed by residues Met120 (H3), Pro214 (H4) and Trp255 (H5). A low-occupancy hydrogen bond is formed between the Ace CO and Tyr121 NH. The Phe side-chain participates in a predominantly hydrophobic cluster, comprised of residues Leu117 (H3) and the non-polar moiety of Arg206 (H5) on one side, and Tyr121 (H3), Ala164 (H4), Leu167 (H4), Leu209 (H5), Trp213 (H5), on the opposite side.

The Orn2 mainchain carbonyl group forms a hydrogen bond with the NH1/2 atoms of Arg206. This hydrogen bond is fully retained in the simulations of mode 22 (100% occupancy), and is the strongest polar interaction between the N-terminal moiety of PMX53 (residues 1–3) and C5aR. An analogous interaction has been proposed to form in the C5aR:C5a complex, between the Arg206 side-chain and the C-terminal carboxylate of C5a [58]; in the Nikiforovich model of the C5aR:C5a complex [25] the Arg206 side-chain interacts with the C-terminal carboxylate of C5a. On the other hand, the mutation Arg206Ala does not abrogate antagonism in linear and cyclic hexapeptides [5]. It is plausible that the binding mode of these antagonists is somewhat different in the absence of the Arg206 side-chain.

Residue Pro3 is buried between helices H5 and H6, interacting on the H5 side with Ala203, the non-polar moiety of Arg206 and Leu207, and on the H6 side with residues Tyr258, Met265, and to a lesser extent Gly262.

The dCha4 carbonyl forms a medium occupancy (36%) hydrogen bond with the Arg206 side-chain (H5). The dCha4 side-chain ring participates in a cluster of “T-shape” interactions with residues Trp5 (PMX53), Phe172 (H4) and His 194 (EC2). It forms additional nonpolar interactions with the non-polar part of Glu199 (EC2), and residues Val202, Ala203 (H5) and Met 265 (H6).

The Trp5 ring is located at the membrane interface, near EC2 and helices H3 and H6. Earlier work suggested that this residue binds in the vicinity of Ile116 [5]. The substitution Ile116Ala correlated with the emergence of weak agonist activity in a PMX53 derivative with benzothiazolalanine at position 5 [5], and converted a linear peptide mimic from antagonist to agonist [58]. Based on these results and the observation that PMX53 remains a full antagonist of the mutant C5aR Ile116Ala, Higginbottom *et al*. argued that the activation of C5aR might require interactions between the side-chain at position 5 and residues located more deeply in the binding pocket that Ile116 [5]. In their modeling of the C5aR:PMX53 complex, the proximity between Trp5 and Ile116 was introduced *a priori* as a geometric restraint. The Trp position in binding mode 22 is consistent with these observations and results without any restraints; Trp5 is predicted to bind in a position less buried than Ile116 (in agreement with the PMX53 antagonist activity), and in its vicinity (the average Ile116 CD – Trp5 O distance is 5.4 ± 0.6 Å in the MD simulation of complex 22). Its side-chain forms a hydrogen bond with the His194 side-chain and numerous nonpolar contacts with residues Cys188, Gly189, Val190, Asp191, Glu199. On the other side, it interacts with the side-chain of Met265. Furthermore, it participates in a cluster of “T-shape” interactions involving residues Phe172 (H4), His 194 (EC2) and dCha4. Trp5 Cα and Cβ atoms interact with the non-polar moiety of Pro113 and to a lesser extent with the side-chain of residue Ile116.

The Arg6 mainchain CO group forms a high-occupancy (98%) hydrogen bond with the Tyr258 (H6) side-chain; the side-chain forms two persistent salt bridges with the Asp282 (EC3) and Asp191 (EC2) carboxylates, a frequent (56% occupancy) hydrogen bond with Leu 278 O (EC3) and a less frequent (18% occupancy) hydrogen bond with Met265 S (H6). These interactions constitute the strongest polar interaction free energy components of mode 22 (Figures 4 and 5). The Arg6-Asp282 interaction is in agreement with earlier studies, which have shown that Asp282 interacts with the Arg74 residue of C5a, and the C-terminal Arg of peptide analogs [5,59,60]. Furthermore, Arg6 makes numerous non-polar contacts with the nonpolar moieties of Tyr258, Met265, Leu278, Leu282 of EC3, as well as nearby residues Cys287 and Val286 of H7. The mainchain Cα atom is placed in proximity to side-chain groups of Pro113 and Ile116 of H3.

### Comparison of binding modes 6, 3, 30 and 22

The next two best binding modes were 6 and 3, with corresponding binding free energies of −166 kcal/mol (~9-10 kcal/mol larger relative to complex 22; see Additional file 1: Figure S1).

**Figure 6:**
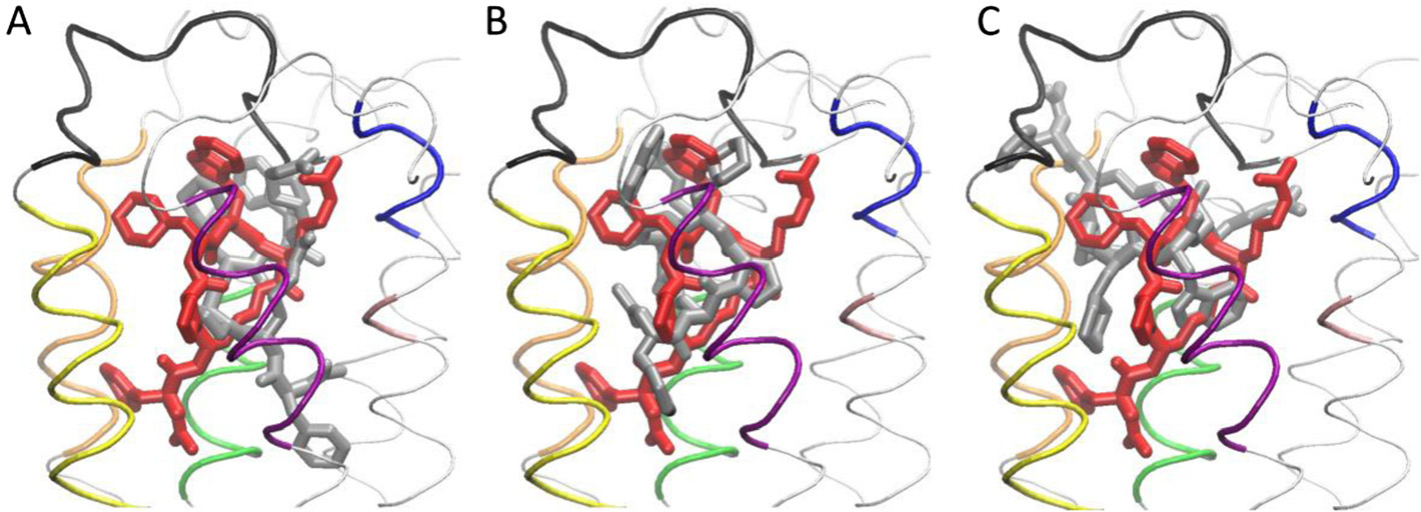
**Comparison of PMX53 structures from the MD simulations of modes 6 (gray; panel A), 3 (gray; panel B), and 30 (gray; panel C) superposed against the lowest binding free energy conformation of complex 22 (red; all panels).** The conformations shown have the lowest binding free energy among all snapshots of the corresponding trajectory (in the MM-PBSA approximation). The structures are aligned using the mainchain transmembrane atoms of C5aR. All ligand hydrogen atoms are omitted for clarity. The C5aR segments interacting with the ligand are shown in different colors, in tube representation; the coloring scheme is the same as in Figure 3.

Figure 6A displays a superposition of representative conformations from the MD runs of modes 6 and 22. The ligand mainchains are approximately at the same distance from the membrane/water interface. They are somewhat displaced relative to each other in a direction parallel to the interface (xy plane), and are rotated by ca. 60° around the perpendicular axis Oz, and to a smaller extent around axes vertical to planes xz and yz. The RMSD between the ligand conformations with the lowest binding free energies for modes 6 and 22 is ~ 4.5 Å (ligand atoms N, Cα, and C).

In the simulations of binding mode 22, PMX53 forms two stable intermolecular salt bridges (Arg6-Asp282 and Arg6-Asp191). In the simulations of mode 6, Arg6 makes the first bridge in segment 0–9.5 ns, and the latter in the second half of the simulation (not shown). Numerous new interactions are observed: The Trp5 side-chain forms interactions with the side-chains of residues Cys109, Leu112, Pro113. PMX53 segment 1–3 interacts with helix H6 in mode 6, and helices H3 and H5 in mode 22.

Figure 6B displays a superposition of representative PMX53 conformations from binding modes 3 and 22; the RMSD between the conformations with the lowest binding free energies is ~ 4.4 Å (ligand atoms N, Cα, and C). The peptide binds at a similar distance from the membrane interface as in modes 6 and 22, and at a similar orientation (with its N-terminal end pointing toward the membrane center). Compared to mode 22, it is rotated by ~90° around the z-axis. As a result, the PMX53 side-chains point toward opposite directions and the moiety 1–5 interacts mainly with helices H5 and H6. Arg6 forms polar and non-polar interactions with domains E23, H4 and EC2, and a stable salt bridge with Glu199. Interestingly, Higginbottom *et al.* have argued that Glu199 might form an interaction with the terminal Arg side-chain of C5 and peptide mimics, and introduced a Glu 199 – Arg6 salt bridge as a restraint in a C5aR – PMX53 docking study described in Ref. [5] (Figure five(C) of that work) [5]. In our mode 3 this interaction is predicted without any guiding restraint. We note that the extracellular loop EC2 of that Higginbottom model had a more open conformation and the Cys109 – Cys188 disulfide bridge was absent; however, the disulfide bridge is a covalent figure and should still be present in the open-loop conformation.

Figure 6C displays a superposition of representative PMX53 conformations from binding modes 30 and 22. Unlike modes 22, 3 and 6, here the N-terminal end of the ligand is oriented toward the membrane interface and the N-terminal Ac group is positioned near the EC2 loop. In this orientation, the ligand N-terminal Ac group is more amenable to substitutions, in agreement with the experimental observation that the replacement of the Ac group by a range of groups with variable size, hydrophobicity and hydrophilicity does not affect the affinity or antagonist activity of PMX53 against C5aR [61]. Despite the change in orientation, the ligand makes the same two key intermolecular electrostatic interactions observed in mode 22 (see Additional file 1: Figure S2): The Arg6 side-chain forms persistent salt-bridges with Asp282 and Asp191 throughout the simulation, as was observed in mode 22. The Orn2 CO group makes a stable hydrogen bond with the Arg206 side-chain in the segment 9.5 ns – 20 ns. The binding free energy of mode 30 is −155 kcal/mol in the first 9.5 ns and −165 kcal/mol in the last 7.5 ns of the simulation. Interestingly, in mode 30 the Trp5 side-chain is positioned between the side-chains of Ile116 and Val286; this pair has been suggested to form an activation switch for C5aR [58]. The average distances Trp5 CH2 - Ile116 CG2 and Trp5 CH2 - Val286 CG are, respectively, 4.6 ± 1.2 Å, and 5.0 ± 0.6 Å.

#### Comparison of binding mode 22 and the C5a:C5aR complex (Nikiforovich model)

**Figure 7:**
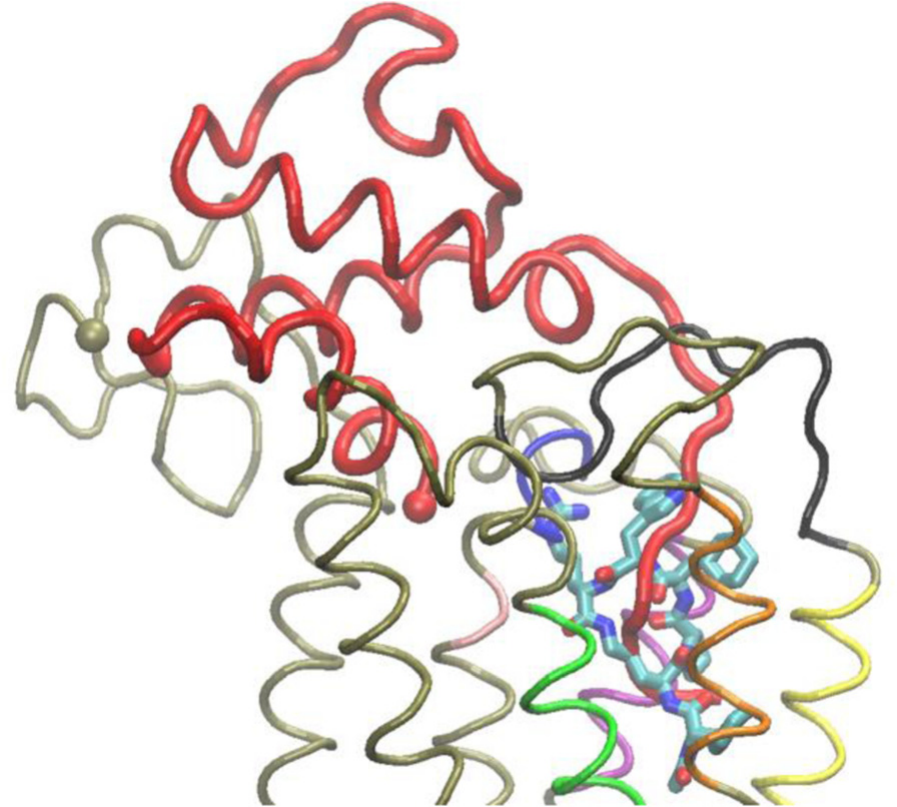
**The C5aR:PMX53 complex (conformation of lowest binding free energy in the simulation of binding mode 22) is superposed against the Nikiforovich model **[25]** for the C5aR:C5a complex.** The C5aR mainchain (mode 22) is indicated by a thin tan tube, and the Cα atom of the first N-terminal end residue of the simulation system (8^th^ residue of C5aR) by a vdW sphere. Interacting protein domains are colored in different colors; the coloring scheme is as in Figure 3. C5a is displayed as a red tube and the Cα atom of the 1^st^ residue is indicated by a vdW sphere. PMX53 is shown in licorice, colored by atom type. Hydrogen atoms are omitted for clarity.

A detailed comparison of the promising binding modes for the C5aR:PMX53 complex, predicted in this work, and the Nikiforovich model for the C5aR:C5a complex [25] can help interpret the antagonist activity of PMX53. Figure 7 displays the binding conformations of C5a and PMX53 ligands, obtained by alignment of the intra-membrane C5aR mainchain heavy atoms in the C5aR:C5a complex (Nikiforovich model) and C5aR:PMX53 complex (mode 22) with the program SuperPose [62]. Additional file 1: Figure S3A-D display maps of side-chain and mainchain contacts in the C5a:C5aR Nikiforovich model; [25] intermolecular hydrogen bonds in the C5a:C5aR complex are presented in Additional file 1: Figure S3E.

According to the superposition presented in Figure 7, PMX53 blocks the entry of the C5a C-terminal into the transmembrane domain of C5aR, by occupying the position in which the C-terminal domain of C5a binds and promotes signaling. PMX53 does not directly act as a mimetic of the C5a C-terminal, as PMX53 is cyclic and the C-terminal domain of C5a is linear. Upon superposition of the PMX53 and C5a in complex with C5aR, the N-terminal end of PMX53 coincides with the C-terminal end of C5a, while the 71–73 C-terminal domain of C5a is placed between the PMX53 3–5 backbone moiety on one site and the Orn2 side-chain moiety on the other site. Also, the backbone of C5a residues Asp369 and Met370 structurally coincide, upon superposition, with the side-chain of PMX53 residue Trp5. The C5aR residue Asp191 makes a salt bridge with PMX53 residue Arg6 in mode 22, and with C5a residue Lys68 in the C5aR:C5a complex. Furthermore, the first four PMX53 residues interact strongly with H5 residues Ala203, Arg206, Leu207, Leu209; these interactions could compete with interactions within the C5a 69–74 residue moiety, and particularly with the salt bridge between Arg206 and the C-terminal carboxyl group of Arg74. Last but not least, H6 residues Gly262 and Met265 interact with C5a segment 67–70 and Arg74 in the Nikiforovich model; these interactions may also be blocked by interactions of C5aR with Orn2, Pro3, dCha4 and Trp5 of PMX53.

## Conclusion

In the present work we construct structural models for the complex of membrane-embedded C5aR and its antagonist peptide PMX53 via a computational framework that combines conformational sampling for both receptor and ligand, docking and filtering of conformations by structural and energetic criteria. A large body of experimental results suggests that the C5aR binding site of the antagonist cyclic hexapeptide PMX53 is at or near the TM binding site of the C5aR agonist peptide C5a [5]. The key PMX53 residue Trp5, an important determinant of antagonism, is likely to be positioned near Ile116, a residue implicated in interactions with the PMX family of peptides and possibly the activation of C5aR [58]. Furthermore, Arg6 has been shown to interact with Asp282 [5]. The most promising binding mode (22) and several other modes of low binding free energy reproduce both interactions. An additional interaction is observed in several high-affinity modes, between the mainchain CO group of Orn2 and the Arg206 side-chain. An analogous interaction has been proposed to form in the C5aR:C5a complex, between the Arg206 side-chain and the C-terminal carboxylate of C5a [58] and is present in the Nikiforovich model of the C5aR:C5a complex [25].

Notably, the aforementioned interactions are predicted without imposing any *a priori* geometric constraints in the initial conformations of the complexes. This successful outcome lies in the use of a multi-step computational framework, which included the generation of representative receptor and ligand conformations by long MD simulations with high quality implicit-membrane models, the docking of a large combination of receptor and ligand conformations, the comprehensive filtering of several hundred thousand conformations for the complex by structural and free energy criteria, and the re-evaluation of the most promising binding modes by additional MD simulations and binding free energy calculations. 2. Membrane effects were introduced by high-quality implicit-membrane models [29-31], which enabled both the rapid generation of representative structures and the estimation of their binding affinities. Therefore, the computational framework used here is capable of addressing the challenges in generating reliable structural models for GPCR protein-ligand complexes of unknown structure. While, the MM-PBSA and MM-GBSA methods yield large binding free energy values [53,54,63], their use in the specific computational protocol of this study proved useful, with regard to identifying the lowest binding free energy, and thus, the most promising PMX53:C5aR binding mode. The large affinity free energies are partly attributed to the omission of the protein, ligand and complex configuration entropy contributions to binding; due to energy-entropy compensation, when these terms are included in the calculation, they are expected to yield significantly smaller total free energies [64]. These entropic terms are associated with large uncertainties and are expected to cancel out to a large extent in the relative affinities of different binding modes. Therefore, their omission is not expected to be important at the level of accuracy of the present calculation, which identifies most promising binding modes. In a similar fashion, the membrane MM-PBSA and/or MM-GBSA approximations were used to identify the most promising binding modes of a dual tropic HIV-1 gp120 V3 loop in complex with CXCR4 and CCR5, CXCL12 (SDF-1α) in complex with CXCR4 and CCL5 (RANTES) in complex with CCR5; the computationally derived structures were in exceptional agreement with experimental findings [65-68].

The structure of membrane-embedded C5aR:PMX53 complex and its dynamic features, presented here, will serve as a template for biopharmaceutical discovery of peptide, peptidomimetic, and organic compound antagonists of C5aR for targeting of complement-mediated autoimmune and inflammatory diseases. Such antagonists may have superior ADMET (absorption, distribution, metabolism, excretion, and toxicity) properties compared to PMX53 and therefore be more amenable to clinical development. Also, the methodology developed for the generation of the structure of membrane-embedded C5aR:PMX53 prepares the grounds for the generation of similar models for the homologous receptors C3aR and C5L2 with their respective endogenous ligands, as well as agonists and antagonists. C5aR, C3aR, and C5L2 have common as well as distinct structural features and biological functions. Comparative studies at atomic resolution will shed light on the underlying structural, physicochemical, and dynamic properties that mediate the similarity and variability in their structures, dynamics, binding properties, and biological functions.

Although the database of solved GPCR structures is slowly but steadily increasing, and there are more structural templates available today for computational modeling than previously, still there is no structural information for the vast majority of GPCRs; and structural information on GPCR-ligand binding at atomic resolution is rare. The computational framework presented here can be of wide use for the development of GPCR-ligand structural models in membrane environments. Such models will be useful in providing the structural basis for mechanistic studies of the interactions between GPCRs and ligands, and their effects in intra-membrane and intra-cellular dynamics that drive the selection of intra-cellular activation pathways. In addition, such models will be useful in knowledge-based biopharmaceutical discovery.

### Ethics statement

This research does not involve human subjects, human material, or human data.

## Competing interests

The authors declare that they have no competing interests.

## Authors’ contributions

DM and GA designed and coordinated the research; PT and CAK performed the research and analysis; DM, GA, PT, CAK, GVN and TMW discussed the results. GA and PT wrote the manuscript. DM, CAK, TMW and GVN contributed to the manuscript. All authors read and approved the final manuscript.

## Supplementary Material

Additional file 1: Figure S1 Average MM-PBSA binding free energies for the 35 most promising modes of the C5aR:PMX53 complex. **Figure S2.** Structure of the C5aR:PMX3 binding site for mode 30. **Figure S3** (3 pages). Protein – ligand contact maps for the homology-based structure of the Nikiforovich C5aR:C5a model. Click here for file

## References

[B1] Manthey HD Woodruff TM Taylor SM Monk PN Complement component 5a (C5a) Int J Biochem Cell Biol 2009 41 2114 2117 19464229 10.1016/j.biocel.2009.04.005

[B2] Monk PN Scola A-M Madala P Fairlie DP Function, structure and therapeutic potential of complement C5a receptors British J Pharm 2007 152 429 448 10.1038/sj.bjp.0707332 PMC2050825 17603557

[B3] Klos A Wende E Wareham KJ Monk PN International union of basic and clinical pharmacology. LXXXVII. Complement peptide C5a, C4a, and C3a receptors Pharm Rev 2013 65 500 543 23383423 10.1124/pr.111.005223

[B4] Li R Coulthard LG Wu MC Taylor SM Woodruff TM C5L2: a controversial receptor of complement anaphylatoxin, C5a FASEB J 2013 27 855 864 23239822 10.1096/fj.12-220509

[B5] Higginbottom A Cain SA Woodruff TM Proctor LM Madala PK Tyndall JDA Taylor SM Fairlie DP Monk PN Comparative Agonist/Antagonist responses in mutant human C5a receptors define the ligand binding site J Biol Chem 2005 280 17831 17840 15661745 10.1074/jbc.M410797200

[B6] Woodruff TM Strachan AJ Dryburgh N Shiels IA Reid RC Fairlie DP Taylor SM Antiarthritic activity of an orally active C5a receptor antagonist against antigen-induced monarticular arthritis in the rat Arthritis Rheum 2002 46 2476 2485 12355496 10.1002/art.10449

[B7] Markiewski MM DeAngelis RA Benencia F Ricklin-Lichtsteiner SK Koutoulaki A Gerard C Coukos G Lambris JD Modulation of the anti-tumor immune response by complement Nat Immunol 2008 9 1225 1235 18820683 10.1038/ni.1655 PMC2678913

[B8] Woodruff TM Ager RR Tenner AJ Noakes PG Taylor SM The role of the complement system and the activation fragment C5a in the central nervous system Neruomol Med 2010 12 179 192 10.1007/s12017-009-8085-y 19763906

[B9] Woodruff TM Nandakumar KS Tedesco F Inhibiting the C5–C5a receptor axis Mol Immunol 2011 48 1631 1642 21549429 10.1016/j.molimm.2011.04.014

[B10] Wong KA Finch AM Pierens GK Craik DJ Taylor SM Fairlie DP Small molecular probes for G-protein-coupled C5a receptors: conformationally constrained antagonists derived from the C terminus of the human plasma protein C5a J Med Chem 1998 41 3417 3425 9719594 10.1021/jm9800651

[B11] Finch AM Wong AK Paczkowski NJ Wadi SK Craik DJ Fairlie DP Taylor SM Low-molecular-weight peptidic and cyclic antagonists of the receptor for the complement factor C5a J Med Chem 1999 42 1965 1974 10354404 10.1021/jm9806594

[B12] Woodruff TM Strachan AJ Sanderson SD Monk PN Wong AK Fairlie DP Taylor SM Species dependence for binding of small molecule agonist and antagonists to the C5a receptor on polymorphonuclear leukocytes Inflammation 2001 25 171 177 11403208 10.1023/a:1011036414353

[B13] Morgan M Bulmer AC Woodruff TM Proctor LM Williams HM Stocks SZ Pollitt S Taylor SM Shiels IA Pharmacokinetics of a C5a receptor antagonist in the rat after different sites of enteral administration Eur J Pharm Sci 2008 33 390 398 18337070 10.1016/j.ejps.2008.01.009

[B14] Granier S Kobilka B A new era of GPCR structural and chemical biology Nat Chem Biol 2012 8 670 673 22810761 10.1038/nchembio.1025 PMC4031315

[B15] Rasmussen S DeVree BT Zou Y Kruse AC Chang KY Kobilka TS Thian FS Chae PS Pardon E Calinski D Mathiesen JM Shah STA Lyons JA Caffrey M Gellman SH Steyaert J Skiniotis G Weis WI Sunahara RK Kobilka BK Crystal structure of the β2 adrenergic receptor-Gs protein complex Nature 2011 477 549 555 21772288 10.1038/nature10361 PMC3184188

[B16] Wu H Wacker D Mileni M Katritch V Han GW Vardy E Liu W Thompson AA Huang X-P Carroll FI Mascarella SW Westkaemper RB Mosier PD Roth BL Cherezov V Stevens RC Structure of the human κ-opioid receptor in complex with JDTic Nature 2012 485 327 333 22437504 10.1038/nature10939 PMC3356457

[B17] Thompson AA Liu W Chun E Katritch V Wu H Vardy E Huang X-P Trapella C Guerrini R Calo G Roth BL Cherezov V Stevens RC Structure of the Nociceptin/Orphanin FQ receptor in complex with a peptide mimetic Nature 2012 485 395 401 22596163 10.1038/nature11085 PMC3356928

[B18] Haga K Kruse AC Asada H Yurugi-Kobayashi T Shiroishi M Zhang C Weis WI Okada T Koblika BK Haga T Kobayashi T Structure of the human M2 muscarinic acetylcholine receptor bound to an antagonist Nature 2012 482 547 551 22278061 10.1038/nature10753 PMC3345277

[B19] Kruse AC Hu J Pan AC Arlow DH Rosenbaum DM Rosemond E Green HF Liu T Chae PS Dror RO Shaw DE Weis WE Wess J Kobilka BK Structure and dynamics of the M3 muscarinic acetylcholine receptor Nature 2012 482 552 556 22358844 10.1038/nature10867 PMC3529910

[B20] Dror RO Dirks RM Grossman JP Xu H Shaw DE Biomolecular simulation: a computational microscope for molecular biology Annu Rev Biophys 2012 41 429 452 22577825 10.1146/annurev-biophys-042910-155245

[B21] Michino M Chen J Stevens RC Brooks CL III FoldGPCR: structure prediction protocol for the transmembrane domain of G protein-coupled receptors from class A Proteins 2010 78 2189 2201 20544957 10.1002/prot.22731 PMC2933064

[B22] Thayer AM Improving peptides Chem Eng News 2011 89 22 13 20

[B23] Nikiforovich GV Marshall GR Modeling flexible loops in the dark-adapted and activated states of rhodopsin, a prototypical G-protein-coupled receptor Biophys J 2005 89 3780 3789 16199504 10.1529/biophysj.105.070722 PMC1366946

[B24] Matsumoto ML Narzinski K Kiser PD Nikiforovich GV Baranski TJ A comprehensive structure-function map of the intracellular surface of the human C5a receptor. I. Identification of critical residues J Biol Chem 2007 282 3105 3121 17135254 10.1074/jbc.M607679200

[B25] Nikiforovich GV Marshall GR Baranski TJ Modeling molecular mechanisms of binding of the anaphylatoxin C5a to the C5a receptor Biochemistry 2008 47 3117 3130 18275159 10.1021/bi702321a

[B26] Hagemann IS Miller DL Kico JM Nikiforovich GV Baranski TJ Structure of the complement factor 5a receptor-ligand complex studied by disulfide trapping and molecular modeling J Biol Chem 2008 283 7763 7775 18195008 10.1074/jbc.M709467200

[B27] Nikiforovich GV Baranski TJ Structural mechanisms of constitutive activation in the C5a receptors with mutations in the extracellular loops: molecular modeling study Proteins 2012 80 71 80 21960464 10.1002/prot.23162 PMC3240690

[B28] Zhang L Mallik B Morikis D Structural study of Ac-Phe-[Orn-Pro-dCha-Trp-Arg], a potent C5a recptor antagonist by NMR Peptide Sci 2008 90 803 815 10.1002/bip.21099 18846566

[B29] Im W Feig M Brooks CL III An implicit membrane generalized born theory for the study of structure, stability, and interactions of membrane proteins Biophys J 2003 85 2900 2918 14581194 10.1016/S0006-3495(03)74712-2 PMC1303570

[B30] Lee MS Feig M Salzbury FR Brooks CL III New analytic approximation to the standard molecular volume definition and its application to generalized Born calculations J Comp Chem 2003 24 1348 1356 12827676 10.1002/jcc.10272

[B31] Tanizaki S Feig M A generalized Born formalism for heterogeneous dielec- tric environments: application to the implicit modeling of biological membranes J Chem Phys 2005 122 124706 15836408 10.1063/1.1865992

[B32] Feig M Brooks CL III Recent advances in the development and application of implicit solvent models in biomolecule simulations Curr Op Struct Biol 2004 14 217 224 10.1016/j.sbi.2004.03.009 15093837

[B33] Yuzlenko O Lazaridis T Membrane protein native state discrimination by implicit membrane models J Comp Chem 2013 34 731 738 23224861 10.1002/jcc.23189 PMC3584241

[B34] MacKerell AD Bashford D Bellott M Dunbrack RL Evanseck JD Field MJ Fischer S Gao J Guo H Ha S Joseph-McCarthy D Kuchnir L Kuczera K Lau FTK Mattos C Michnick S Ngo T Nguyen DT Prodhom B Reiher WE Roux B Schlenkrich M Smith JC Stote R Straub J Watanabe M Wiorkiewicz-Kuczera J Yin D Karplus M All-atom empirical potential for molecular modeling and dynamics studies of proteins J Phys Chem B 1998 102 3586 3616 24889800 10.1021/jp973084f

[B35] Mackerell AD Jr Feig M Brooks CL III Extending the treatment of backbone energetics in protein force fields: limitations of gas- phase quantum mechanics in reproducing protein conformational distributions in molecular dynamics simulations J Comput Chem 2004 25 1400 1415 15185334 10.1002/jcc.20065

[B36] Brooks BR Brooks CL III Mackerell AD Nilsson L Petrella RJ Roux B Won Y Archontis G Bartels C Boresch S Caflisch A Caves L Cui Q Dinner AR Feig M Fischer S Gao J Hodoscek M Im W Kuczera K Lazaridis T Ma J Ovchinnikov V Paci E Pastor RW Post CB Pu JZ Schaefer M Tidor B Venable RM CHARMM: the biomolecular simulation program J Comput Chem 2009 30 1545 1614 19444816 10.1002/jcc.21287 PMC2810661

[B37] Karpen ME Tobias DJ Brooks CL III Statistical clustering techniques for analysis of long molecular dynamics trajectories. I: analysis of 2.2 ns trajectories of YPGDV Biochemistry 1993 32 412 420 8422350 10.1021/bi00053a005

[B38] Carpenter GA Grossberg S ART-2: self-organization of stable category recognition codes for analog input patterns Appl Opt 1987 26 4919 4930 20523470 10.1364/AO.26.004919

[B39] Nikiforovich GV Taylor CM Marshall GR Modeling of the complex between transducin and photoactivated rhodopsin, a prototypical G-protein-coupled receptor Biochemistry 2007 46 4734 4744 17397191 10.1021/bi700185p

[B40] The UniProt Consortium Update on activities at the Universal Protein Resource (UniProt) in 2013 Nucleic Acids Res 2013 41 D43 D47 23161681 10.1093/nar/gks1068 PMC3531094

[B41] Lomize MA Lomize LA Pogozheva ID Mosberg HI OPM: orientations of proteins in membranes database Bioinformatics 2006 22 623 625 16397007 10.1093/bioinformatics/btk023

[B42] Ryckaert JP Ciccotti G Berendsen HJC Numerical integration of the cartesian equations of motion of a system with constraints: molecular dynamics of n-alkanes J Comput Phys 1997 23 327 341

[B43] Jaccard P Étude comparative de la distribution florale dans une portion des Alpes et des Jura Bulletin de la Société Vaudoise des Sciences Naturelles 1901 37 547 579

[B44] Jain A Murty M Flynn P Data clustering: a review ACM Comput Surv 1999 31 264 323

[B45] R Development Core Team R: A language and environment for statistical computing R Foundation for Statistical Computing 2011 Vienna, Austria: R Development Core Team

[B46] Grant BJ Rodrigues APC ElSawy KM McCammon JA Caves LSD Bio3D. A package for the comparative analysis of protein structures Bioinformatics 2006 22 2695 2696 16940322 10.1093/bioinformatics/btl461

[B47] Lang PT Brozell SR Mukherjee S Pettersen EF Meng EC Thomas V Rizzo RC Case DA James TL Kuntz ID DOCK 6: combining techniques to model RNA-small molecule complexes RNA 2009 15 1219 1230 19369428 10.1261/rna.1563609 PMC2685511

[B48] Richards FM Areas, volumes, packing, and protein structure Ann Rev Biophys Bioeng 1977 6 151 176 326146 10.1146/annurev.bb.06.060177.001055

[B49] Massova I Kollman PA Combined molecular mechanical and con- tinuum solvent approach (MM-PBSA/GBSA) to predict ligand binding Perspect Drug Discov Des 2000 18 113 135

[B50] Pearlman DA Evaluating the molecular mechanics Poisson-Boltzmann surface area free energy method using a congeneric series of ligands to p38 MAP kinase J Med Chem 2005 48 7796 7807 16302819 10.1021/jm050306m

[B51] Hayes JM Archontis G Wang L MM-GB (PB) SA Calculations of Protein-Ligand Binding Free Energies, Molecular Dynamics - Studies of Synthetic and Biological Macromolecules Tech, Chapter 9 2012 171 190

[B52] Seeber M Felline A Raimondi F Muff S Friedman R Rao F Caflisch A Fanelli F Wordom: a user-friendly program for the analysis of molecular structures, trajectories, and free energy surfaces J Comp Chem 2011 32 1183 1194 21387345 10.1002/jcc.21688 PMC3151548

[B53] Tamamis P Morikis D Floudas CA Archontis G Species specificity of the complement inhibitor compstatin investigated by all-atom molecular dynamics simulations Proteins 2010 78 2655 2667 20589629 10.1002/prot.22780 PMC3138065

[B54] Tamamis P Pierou P Mytidou C Floudas CA Morikis D Archontis G Design of a modified mouse protein with ligand binding properties of its human analog by molecular dynamics simulations: the case of C3 inhibition by compstatin Proteins 2011 79 3166 3179 21989937 10.1002/prot.23149 PMC3193182

[B55] Findlay JB Pappin DJ The Opsin family of proteins Biochem J 1986 238 625 642 2948499 10.1042/bj2380625 PMC1147185

[B56] Nikiforovich GV Marshall GR Baranski TJ Simplified modeling approach suggests structural mechanisms for constitutive activation of the C5a receptor Proteins 2011 79 787 802 21287612 10.1002/prot.22918 PMC3072438

[B57] Nikiforovich GV Taylor CM Marshall G Baranski TJ Modeling the possible conformations of the extracellular receptors in G-protein-coupled receptors Proteins 2010 78 271 285 19731375 10.1002/prot.22537 PMC2795062

[B58] Gerber BO Meng EC Doetsch V Baranski TJ Bourne HR An activation switch in the ligand binding pocket of the C5a receptor J Biolog Chem 2001 276 3394 3400 10.1074/jbc.M007748200 11062244

[B59] Cain SA Coughlan T Monk PN Mapping the ligand-binding site on the C5a receptor: arginine 74 of C5a contacts aspartate 282 of the C5a receptor Biochemistry 2001 40 14047 14052 11705397 10.1021/bi011055w

[B60] Cain SA Higginbottom A Monk PN Characterisation of C5a receptor agonists from phage display libraries Biochem Pharmacol 2003 66 1833 1840 14563493 10.1016/s0006-2952(03)00473-8

[B61] March DR Proctor LM Stoermer MJ Sbaglia R Abbenante G Reid CR Woodruff TM Wadi K Paczkowski N Tyndall JDA Taylor SM Fairlie D Potent cyclic antagonists of the complement C5a receptor on human polymorphonuclear leukocytes. Relationships between structure and activity Molec Pharmacol 2004 65 868 879 15044616 10.1124/mol.65.4.868

[B62] Maiti R Van Domselaar GH Zhang H Wishart DS SuperPose: a simple server for sophisticated structural superposition Nucleic Acids Res 2004 32 Web Server issue W590W594 15215457 10.1093/nar/gkh477 PMC441615

[B63] Tamamis P de Victoria López A Gorham RD Jr Bellows-Peterson ML Pierou P Floudas CA Morikis D Archontis G Molecular dynamics in drug design: new generations of compstatin analogs Chem Biol Drug Des 2012 79 703 718 22233517 10.1111/j.1747-0285.2012.01324.x PMC3319835

[B64] Gilson MK Zhou H-X Calculation of protein-ligand binding affinities Annu Rev Biophys Biomol Struct 2007 36 21 42 17201676 10.1146/annurev.biophys.36.040306.132550

[B65] Tamamis P Floudas CA Molecular recognition of CXCR4 by a dual tropic HIV-1 gp120 V3 loop Biophys J 2013 105 1502 1514 24048002 10.1016/j.bpj.2013.07.049 PMC3785887

[B66] Tamamis P Floudas CA Molecular recognition of CCR5 by an HIV-1 gp120 V3 Loop PLoS ONE 2014 9 e95767 24763408 10.1371/journal.pone.0095767 PMC3999033

[B67] Tamamis P Floudas CA Elucidating a key component of cancer metastasis: CXCL12 (SDF-1α) binding to CXCR4 J Chem Inf Model 2014 54 1174 1188 24660779 10.1021/ci500069y PMC4004218

[B68] Tamamis P Floudas CA Elucidating a key anti-HIV-1 and cancer-associated axis: the structure of CCL5 (Rantes) in complex with CCR5 Sci Rep 2014 4 5447 24965094 10.1038/srep05447 PMC4894430

