# Supplementary material for: Insights into the mechanism of C5aR inhibition by PMX53 via implicit solvent molecular dynamics simulations and docking
Source: BMC Biophys. 2014 Aug 12;7:5. doi: 10.1186/2046-1682-7-5 (PMC4141665; doi:10.1186/2046-1682-7-5)
Supplement: Additional file 1: Figure S1 — Average MM-PBSA binding free energies for the 35 most promising modes of the C5aR:PMX53 complex. Figure S2. Structure of the C5aR:PMX3 binding site for mode 30. Figure S3 (3 pages). Protein – ligand contact maps for the homology-based structure of the Nikiforovich C5aR:C5a model. [file 2046-1682-7-5-S1.pdf]

Insights into the Mechanism of C5aR Inhibition by PMX53 using Implicit Solvent Molecular Dynamics Simulations and Docking

Phanourios Tamamis[1], Chris A. Kieslich[2], Gregory V. Nikiforovich[3], Trent M. Woodruff[4], Dimitrios Morikis[2], Georgios Archontis[1]

[1]Department of Physics, University of Cyprus, PO 20537, CY1678, Cyprus

[2]Department of Bioengineering, University of California Riverside, CA 92521, USA.

[3]MolLife Design LLC, St. Louis 63141, USA.

[4]School of Biomedical Sciences, the University of Queensland, Queensland, St Lucia, 4072, Australia.

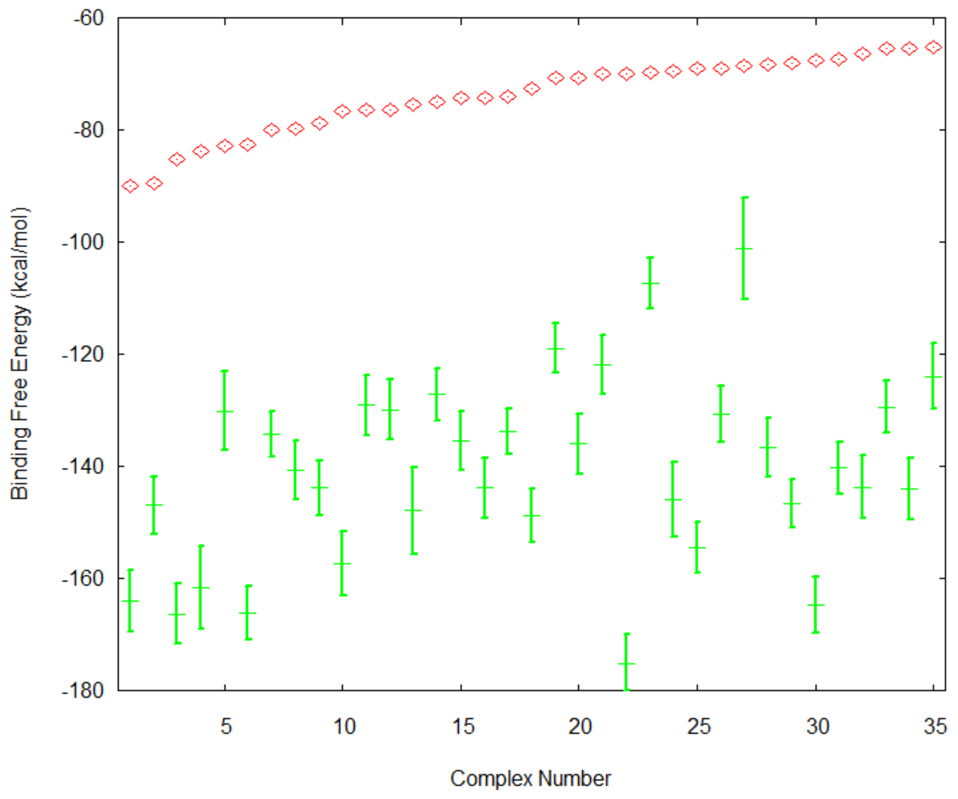

Supplementary Figure 1: Average MM-PBSA binding free energies for the 35 most promising modes of the C5aR:PMX53 complex. The free energies are averaged over implicit-membrane MD simulations with a duration of 7 – 20 ns (see main text). Red diamonds correspond to the MM-PBSA binding free energies of the complex structures before MD simulations. Green crosses correspond to the average MM-PBSA binding free energies of the simulated complexes (see Evaluation of structural models for the C5aR:PMX53 complex by MD simulations, main text). The standard deviation of the MM-PBSA binding free energies within the simulations is presented in green error bars.

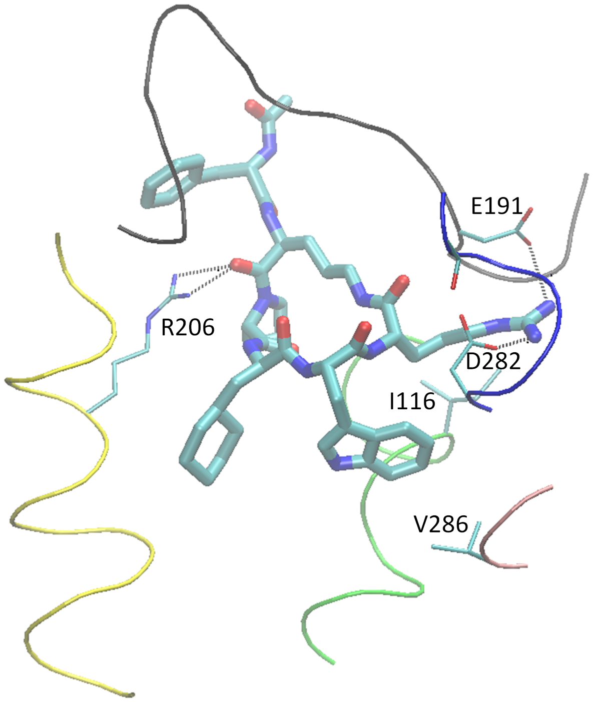

Supplementary Figure 2: Structure of the C5aR:PMX3 binding site for mode 30. The ligand is shown in licorice representation and the surrounding protein is shown as a trace; protein residues forming important interactions with the ligand are shown in licorice representation. The coloring of protein segments is as in Figure 3 of the main text. Protein segments in contact with PMX53 are indicated in the following colors: H3 in green, EC2 in black, H5 in yellow, EC3 in blue, and H7 in pink. Important C5aR:PMX53 interactions are indicated and discussed in the main text.

Supplementary Figure 3: Protein – ligand contact maps for the homology-based structure of the Nikiforovich C5aR:C5a model.[1]

A) Map of Sidechain – Sidechain Contacts. The horizontal and vertical axes contain, respectively, C5aR and C5a residues.

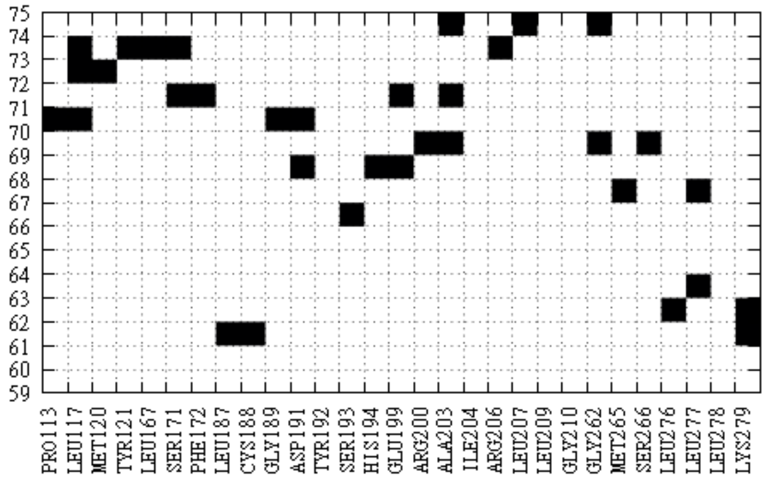

B) Map of Mainchain – Mainchain Contacts. The horizontal and vertical axes contain, respectively, C5aR and C5a residues.

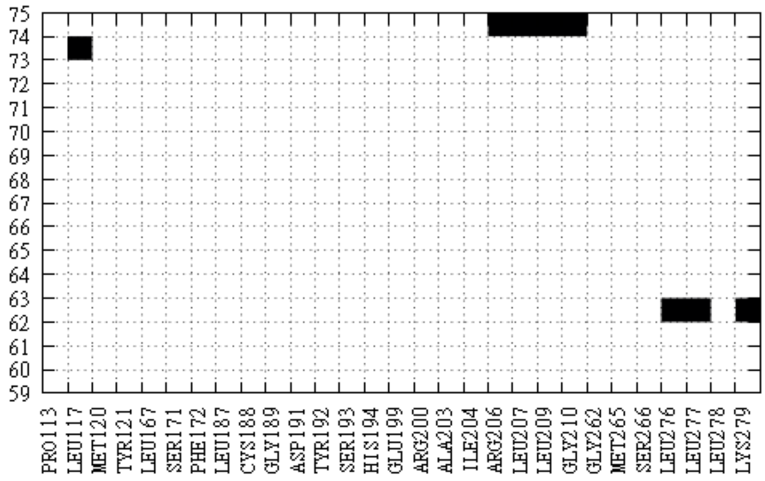

C) Map of C5aR Sidechain – C5a Mainchain Contacts. The horizontal and vertical axes contain, respectively, C5aR and C5a residues.

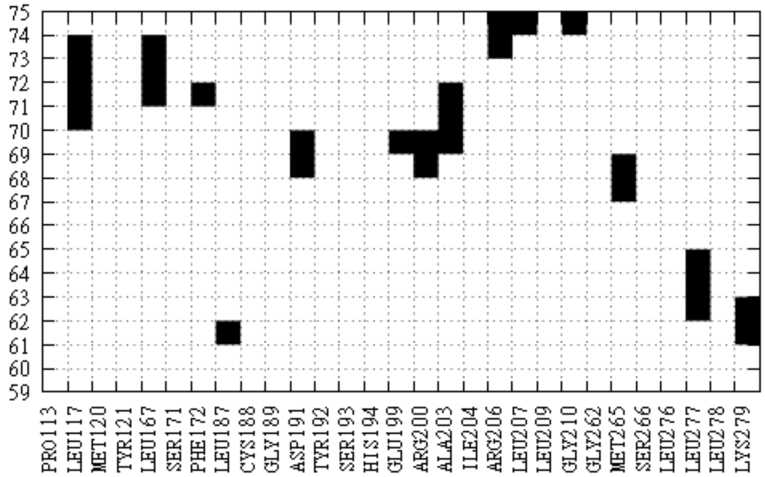

D) Map of C5aR Mainchain – C5a Sidechain Contacts. The horizontal and vertical axes contain, respectively, C5aR and C5a residues.

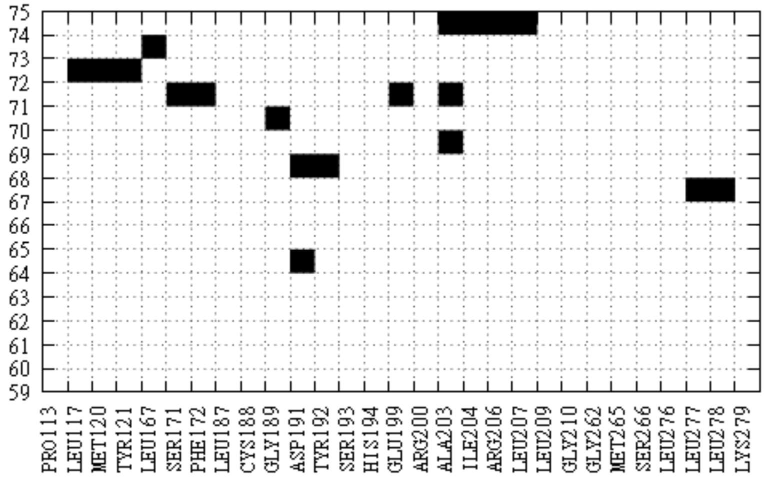

E) Map of C5aR – C5a hydrogen bonds and salt bridges. The horizontal and vertical axes contain, respectively, C5aR and C5a residues.

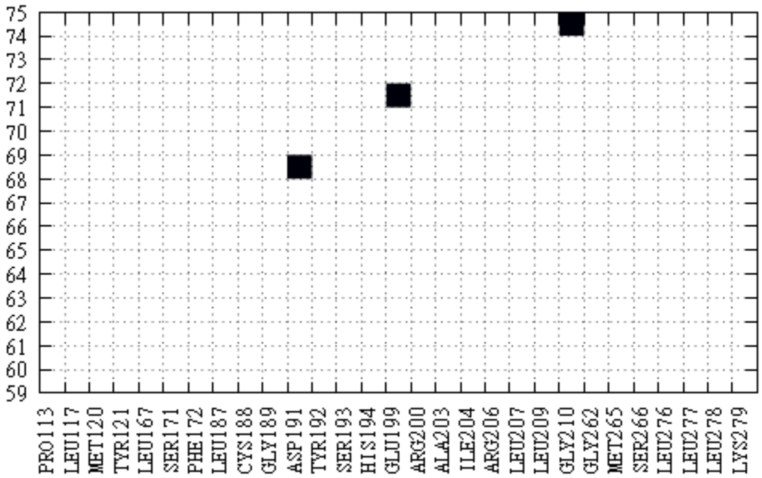

References

1. G. V. Nikiforovich, G. R. Marshall, T. J, Baranski. *Biochemistry* **2008**, *47*, 3117-3130.
